# Clear Cell Papillary Renal Cell Carcinoma: A Potential Mimic of Conventional Clear Cell Renal Carcinoma on Core Biopsy

**DOI:** 10.1155/2015/423908

**Published:** 2015-01-29

**Authors:** Heath Liddell, Anton Mare, Sean Heywood, Genevieve Bennett, Hin Fan Chan

**Affiliations:** ^1^Department of Urology, The Canberra Hospital, Garran, ACT 2605, Australia; ^2^ACT Pathology, The Canberra Hospital, Garran, ACT 2605, Australia

## Abstract

Clear cell papillary renal cell carcinoma (CCP-RCC) is a recently described, relatively uncommon variant of renal cell carcinoma (RCC) with a reported incidence of 4.1%. Thought to only arise in those with end stage renal disease, CCP-RCC is increasingly identified in those without renal impairment. CCP-RCCs have unique morphologic, genetic, and immunohistochemical features distinguishing them from both conventional clear cell renal cell carcinomas and papillary renal cell carcinomas. Immunohistochemically, these tumors are positive for CK7 and negative for CD10 and racemase. This is in contrast to conventional cell renal cell carcinomas (CK7 negative, CD10 positive) and papillary cell carcinomas (CK7, CD10, and racemase positive). These tumours appear to be indolent in nature, with no current documented cases of metastatic spread. We present the case of a 42-year-old female who presented with an incidental finding of a renal mass that on a core biopsy was reported as clear cell carcinoma, Fuhrman grade 1. She subsequently underwent a radical nephrectomy and further histological examination revealed the tumor to be a clear cell papillary renal cell carcinoma, Fuhrman grade 1.

## 1. Introduction

The World Health Organisation in 2004 has classified renal cell carcinoma into the various histological subtypes [1]. The WHO histological classification of renal cell tumors 2004 is as follows:

1. clear cell renal cell carcinoma,
2. multilocular clear cell renal cell carcinoma,
3. papillary renal cell carcinoma (types 1 and 2),
4. chromophobe renal cell carcinoma,
5. carcinoma of the collecting ducts of Bellini,
6. renal medullary carcinoma,
7. Xp11 translocation carcinomas,
8. carcinoma associated with neuroblastoma,
9. mucinous tubular and spindle cell carcinoma,
10. renal cell carcinoma, unclassified,
11. papillary adenoma,
12. oncocytoma.

However in 2000, a new entity began to be recognized having first been reported by Michal et al. [2, 3] and later by Tickoo et al. [4]. More recently the International Society of Urological Pathologists Vancouver Classification recommended including clear cell papillary renal cell carcinoma (amongst others) in the classification [5]. Histologically these tumors consist of a single layer of cells with clear cytoplasm organized in tubular, cystic, and papillary patterns. Immunohistochemically, they are positive for CK7 and negative for CD10 and racemase. They do not exhibit the typical cytogenetic changes associated with either papillary or clear cell renal cell carcinomas [6]. Tickoo et al. initially reported such lesions appearing in patients with end stage renal disease; however subsequent investigators began to identify tumors with equivalent histological and immunohistochemical characteristics in patients without renal impairment [4, 7, 8]. The widespread availability of cross-sectional imaging has led to an increase in the identification of renal masses and a resurgence in the utility of renal biopsy to assist in the characterization of such lesions. There is a growing recognition that chronic kidney disease postradical nephrectomy is associated with increased mortality and therefore a move exists towards nephron sparing surgery or active surveillance in patients with indolent lesions [9]. An appreciation of new histological subtypes of RCC, such as clear cell papillary renal cell carcinoma, and their biological behavior, will assist clinicians to advise patients on the safe and appropriate management of such tumors.

## 2. Case Presentation

We present a case of a 42-year-old female who was found to have an incidental renal mass, which, on histological examination of the excised specimen, was revealed to be a clear cell papillary carcinoma, a relatively uncommon variant of renal cell carcinoma. The patient is a 42-year-old female who presented to her local medical officer with a two-week history of neck pain and radicular like pains extending into her right arm. A CT scan of her cervical spine revealed multilevel intervertebral disc and facet joint degenerative change resulting in neural exit foraminal narrowing. She was noted to have raised inflammatory markers (WCC 27.5, neutrophils 24.8, and CRP 319) and* S. aureus* was isolated on a blood culture. She was commenced on IV antibiotics; however her symptoms failed to improve and she subsequently developed significant lower back pain for which a CT of her lumbar spine was arranged. This revealed no convincing evidence of a lumbar spine infective process; however a 47 × 31 mm solid upper pole mass with an irregular periphery and internal calcification was identified in the left kidney, suspicious for a renal cell carcinoma.

**Figure 1:**
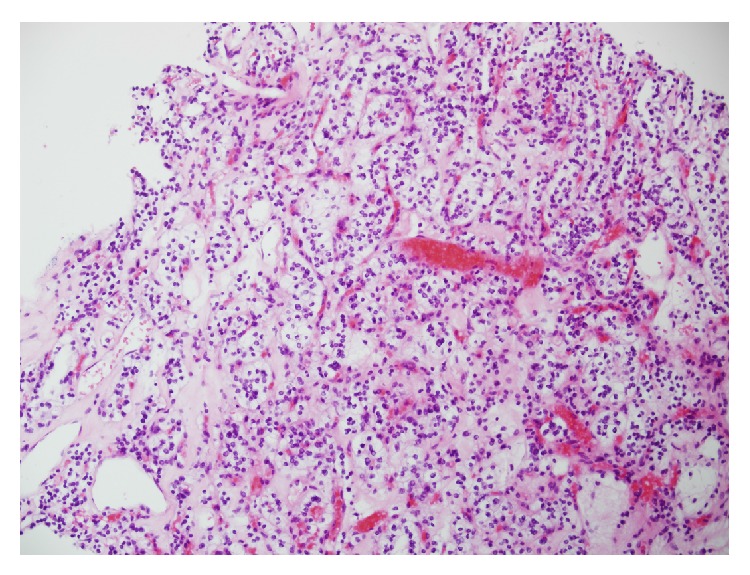
High power of biopsy showing area consistent with clear cell renal cell carcinoma.

The renal mass was further characterized with a triple phase abdominal CT scan and this confirmed a 4 × 4.9 × 4.5 cm heterogenous mass in the upper pole of the left kidney. There was no evidence of left renal vein involvement nor was any radiologically significant lymphadenopathy seen. Her films were reviewed and the decision was made to biopsy the left renal mass to establish whether the lesion was neoplastic or inflammatory. A core biopsy of the left kidney was reported as a Fuhrman grade 1, clear cell renal cell carcinoma (Figure 1). She was then booked for an elective laparoscopic left radical nephrectomy. She underwent this procedure without complication and had an uneventful postoperative course subsequently being discharged home on day four.

Macroscopic examination of the surgical specimen revealed a tumor in the mid portion of the kidney located superior to the renal hilum measuring 48 mm × 40 mm × 46 mm. The tumour was well circumscribed and bulged anteriorly into but not through the renal capsule. The cut surface was variegated cream, light brown, yellow, and dark brown with no areas of necrosis. Focal calcification was seen. Microscopic examination revealed the tumour to consist of uniform cells with clear cytoplasm and low nuclear grade arranged in tubules with small cysts and papillary structures. A single cell layer was seen in these formations. The component cells showed prominent subnuclear cytoplasmic clearing. There were areas of stromal sclerosis with fresh and old haemorrhage, cholesterol crystals, and haemosiderin laden macrophages. Dystrophic calcification was seen in the areas of sclerotic stroma. There were no psammoma bodies. Bundles of smooth muscle were present in the tumour capsule and within the tumour, in areas surrounding the tubular and acinar structures. The tumor cells were strongly positive for CK7 and CK19 and negative for CD10 and racemase. This pattern of staining is in keeping with a clear cell papillary carcinoma rather than a conventional clear cell renal cell carcinoma or papillary cell carcinoma.

## 3. Discussion

Clear cell papillary renal cell carcinoma is a relatively recently recognised variant of renal cell carcinoma and has identical features to other tumours variously classified as clear cell papillary and cystic renal cell carcinoma, clear cell tubulopapillary renal cell carcinoma, renal angiomyoadenomatous tumour, and sporadic renal cell carcinoma with diffuse CK7 positivity [3, 5, 10]. Recent series of nephrectomy specimens reported clear cell papillary carcinoma to be the fourth most common variant of renal cell carcinoma behind clear cell (70%), papillary (16.6%), and chromophobe carcinoma (5.9%) with an incidence of 4.1% [11]. These tumours occur over a wide age range (26–85, mean 60 years) without a predilection for gender [10].

**Figure 2:**
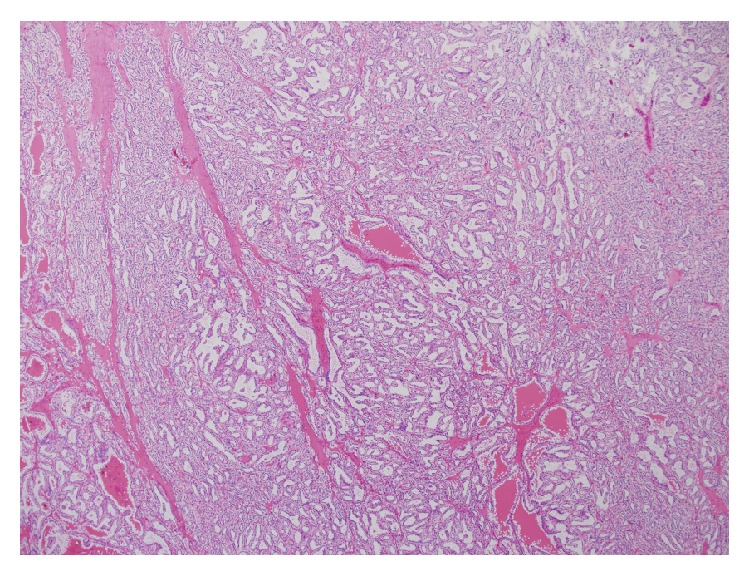
Low power showing dominant tubular architecture.

**Figure 3:**
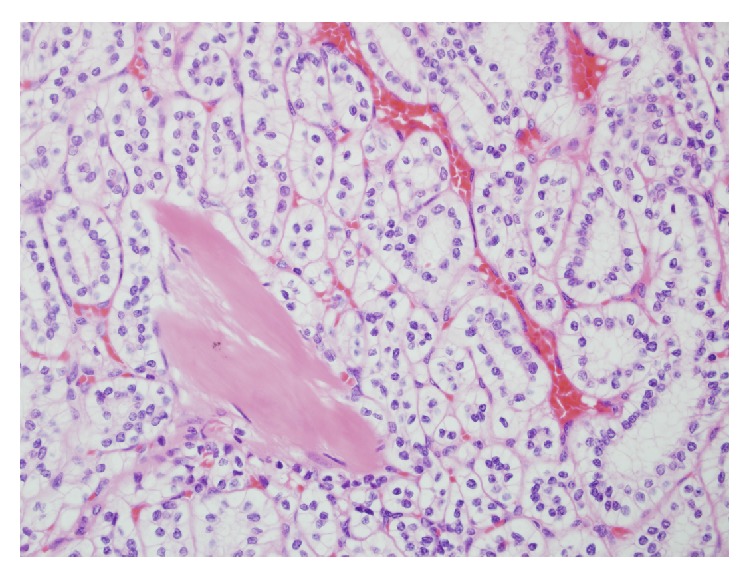
High power showing characteristic subnuclear clearing and low nuclear grade, with smooth muscle bundles in between (lower left corner).

**Figure 4:**
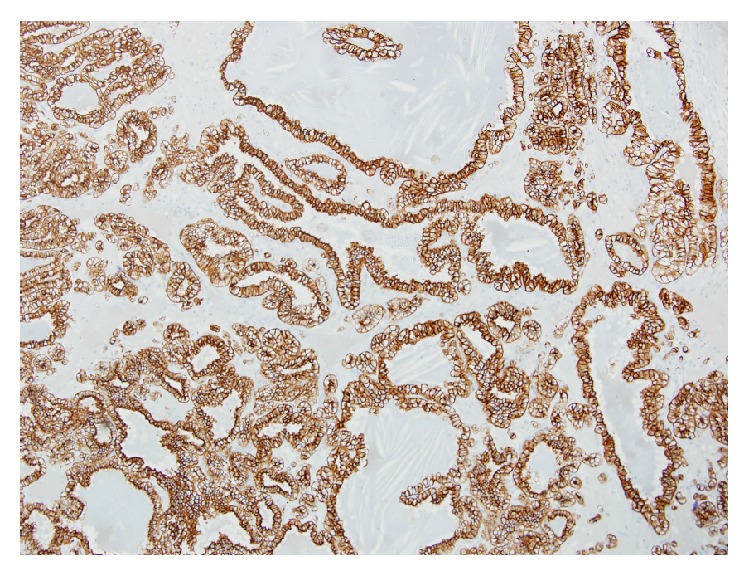
CK7 positive staining highlighting tubular and cystic architecture.

Histologically, these tumors consist of a single layer of cuboidal cells with clear cytoplasm arranged in tubular, cystic, and papillary patterns (Figure 2). Nuclei are typically separated from the base of the cell and the nuclear characteristics are such that all CCP-RCC are Fuhrman grade 1 or 2. Characteristically, the cells have subnuclear vacuoles, similar to the glands of early secretory phase endometrium. Another consistent finding is the presence of smooth muscle bundles within the tumour capsule and scattered throughout the body of the tumour (Figure 3). In contrast to clear cell carcinoma, these tumours are positive for CK7 and unlike papillary carcinoma, they are negative for CD10 and racemase (Figure 4). They do not exhibit gains of chromosome 7 or 17 nor loss of chromosome Y as seen in papillary renal cell carcinoma, nor do they exhibit loss of the chromosome 3p or VHL gene mutations as seen in clear cell carcinoma. They may contain areas with a striking resemblance to conventional clear cell renal cell carcinomas. Given this tumour heterogeneity, the potential for sampling error associated with a small core biopsy specimen means that caution should be exhibited when attempting to make a diagnosis of CCP-RCC on core biopsy. Radiologically, these tumors are indistinct from other renal cell carcinomas, appearing as a solid enhancing heterogenous mass following the administration of IV contrast.

Clinically, over 95% of these tumors are less than 4 cm in size (pT1a) and they do not demonstrate renal vein invasion [10]. To date, there have been no reported cases of metastases associated with these tumors. Given their propensity to be of low grade and stage, and lack of metastatic potential, these tumors are considered to be indolent in nature and perhaps are better classified as clear cell papillary tumours. Given this premise, if a confident histological diagnosis of such a tumour is made on a renal biopsy, they could be actively surveyed particularly in poor surgical candidates with multiple medical comorbidities or in those with preexisting renal disease. Nephrectomy, either partial or radical, offers definitive management of these tumours. Strong consideration should be given to the option of a nephron sparing approach should the size and location of the lesion allow reducing the risk of postoperative chronic kidney disease and its associated sequelae.

In summary, clear cell papillary renal cell carcinoma is an uncommon variant of renal cell neoplasm which while indistinct radiologically from other renal cell tumours has distinct histological and immunohistochemical characteristics. It typically presents as a low grade, low stage tumor and current evidence suggests it has no metastatic potential. This indolent tumor is suitable for active surveillance or a nephron sparing approach should operative management be employed. Pathologists need to be aware of this entity to avoid misdiagnosis, particularly as the biological behavior of this tumour is very different to that of conventional clear cell carcinoma and therefore potentially has different management options. Until well-documented cases of metastasis have been reported, perhaps these neoplasms are more appropriately classified as clear cell papillary tumours.
